# Pencil Beam Scanning Proton Bragg Peak Conformal FLASH in Prostate Cancer Stereotactic Body Radiotherapy

**DOI:** 10.3390/cancers16040798

**Published:** 2024-02-15

**Authors:** Tyler Kaulfers, Grant Lattery, Chingyun Cheng, Xingyi Zhao, Balaji Selvaraj, Hui Wu, Arpit M. Chhabra, Jehee Isabelle Choi, Haibo Lin, Charles B. Simone, Shaakir Hasan, Minglei Kang, Jenghwa Chang

**Affiliations:** 1 Department of Physics and Astronomy, Hofstra University, Hempstead, NY 11549, USA; tkaulfers1@pride.hofstra.edu (T.K.); grant.d.lattery@hofstra.edu (G.L.); 2 Department of Radiation Oncology, Rutgers Cancer Institute of New Jersey, 195 Little Albany Street, New Brunswick, NJ 08901, USA; chingyun.cheng@rutgers.edu; 3 New York Proton Center, 225 E 126th Street, New York, NY 10035, USA; xzhao@nyproton.com (X.Z.); bselvaraj@nyproton.com (B.S.); achhabra@nyproton.com (A.M.C.); ichoi@nyproton.com (J.I.C.); hlin@nyproton.com (H.L.); shasan@nyproton.com (S.H.); 4 Department of Radiation Oncology, The Affiliated Cancer Hospital of Zhengzhou University, Henan Cancer Hospital, Zhengzhou 450008, China; zlyywuhui202@zzu.edu.cn; 5 Northwell, 2000 Marcus Ave, Suite 300, New Hyde Park, NY 11042, USA

**Keywords:** FLASH radiotherapy, prostate cancer, ultra-high dose rate, proton pencil beam scanning, single-energy Bragg peak, intensity-modulated proton therapy, stereotactic body radiotherapy

## Abstract

**Simple Summary:**

Prostate cancer is one of the most diagnosed cancers in men. While it can be successfully treated with radiotherapy (RT), patients often leave treatment with previously healthy organs affected by radiation. This work assessed a novel ultra-high dose rate FLASH proton therapy technique for the treatment of prostate cancers. We were able to generate clinically viable Bragg peak FLASH treatment plans for prostate cancer patients previously treated with conventional proton RT. Feasible prostate treatment quality was observed with all constraints for organs at risk (OARs) being met while maintaining an ultra-high dose rate.

**Abstract:**

Bragg peak FLASH radiotherapy (RT) uses a distal tracking method to eliminate exit doses and can achieve superior OAR sparing. This study explores the application of this novel method in stereotactic body radiotherapy prostate FLASH-RT. An in-house platform was developed to enable intensity-modulated proton therapy (IMPT) planning using a single-energy Bragg peak distal tracking method. The patients involved in the study were previously treated with proton stereotactic body radiotherapy (SBRT) using the pencil beam scanning (PBS) technique to 40 Gy in five fractions. FLASH plans were optimized using a four-beam arrangement to generate a dose distribution similar to the conventional opposing beams. All of the beams had a small angle of two degrees from the lateral direction to increase the dosimetry quality. Dose metrics were compared between the conventional PBS and the Bragg peak FLASH plans. The dose rate histogram (DRVH) and FLASH metrics of 40 Gy/s coverage (V_40Gy/s_) were investigated for the Bragg peak plans. There was no significant difference between the clinical and Bragg peak plans in rectum, bladder, femur heads, large bowel, and penile bulb dose metrics, except for D_max_. For the CTV, the FLASH plans resulted in a higher D_max_ than the clinical plans (116.9% vs. 103.3%). For the rectum, the V_40Gy/s_ reached 94% and 93% for 1 Gy dose thresholds in composite and single-field evaluations, respectively. Additionally, the FLASH ratio reached close to 100% after the application of the 5 Gy threshold in composite dose rate assessment. In conclusion, the Bragg peak distal tracking method can yield comparable plan quality in most OARs while preserving sufficient FLASH dose rate coverage, demonstrating that the ultra-high dose technique can be applied in prostate FLASH SBRT.

## 1. Introduction

Prostate cancer continues to be prevalent in men in the United States [1] and across the globe [2], with a plethora of treatment methods available. Oftentimes, a combination of treatments is required for optimal tumor control [3], and in most cases, the use of radiation therapy (RT) is necessary to improve patient outcomes [4]. The standard treatment for prostate RT is photon-based, but it has limitations. Since photons have no defined range, the mega-voltage (MV) beams used for prostate treatment continue to deliver doses past the target volume, irradiating healthy tissue as well as producing an exit dose. Prostate brachytherapy uses lower energy photons, which have a more conformed dose distribution. However, the use of brachytherapy for prostate treatment has been hindered by the additional quality assurance required for radioactive sources and difficulties in operating room scheduling.

Protons have also been used to treat prostate cancers [4,5]. This is due to their lower exit dose and reduced normal tissue toxicities, allowing for better recovery and results. The effectiveness of protons in radiation therapy has been continuously explored and is now implemented in proton centers across the world for treating various cancers, including prostate cancers. Early reports of clinical outcomes showed that proton therapy (PT) has high efficacy, minimal physician-assessed toxicity, and excellent patient-reported outcomes [6], and it is effective and well-tolerated in prostate cancer patients [7]. The major concern regarding the proton treatment of prostate cancers is its much higher cost in comparison to conventional photon treatment. Given that the cost for proton units has been decreasing, proton therapy might become a cost-effective treatment modality for treating prostate cancers in the near future [8,9].

### 1.1. Background of FLASH Radiotherapy

Research has shown that ultrahigh dose rate radiation therapy, exceeding 40 Gy/s and known as “FLASH” radiation therapy (FLASH-RT), can markedly diminish toxicity in normal tissue while maintaining the effectiveness of conventional dose rate radiotherapy in controlling tumors [10]. Multiple studies [11,12,13,14] on dose rate dependence have been performed for in vitro cell irradiation, but no significant difference in relative biological effect (RBE) was observed. However, in vivo studies of cell irradiation [10] demonstrated a higher survival rate for cells irradiated with this high (>40 Gy/s) dose rate, in comparison to those irradiated at a conventional (<0.6 Gy/s) dose rate. Clinical trials have also been performed on animals [15,16] and superficial lesions [17] that displayed cell healing with minimal scarring, while maintaining similar tumor control as observed in conventional RT.

However, research on FLASH treatments is thus far insufficient, so the cause of the inherent FLASH OAR sparing is yet to be explained well. Favaudon et al. [6] hypothesized that differential responses between normal and tumor tissue are due to the different patterns of DNA damage caused to target cells by FLASH versus conventional irradiations, and a decreased delivery time might provide a longer than normal tissue recovery period. For example, Auer et al. [14] reported that there is an increase of cells in the radioresistant G2 phase, which might allow for better normal cell healing. Spitz et al. [18] suggested that free radicals might play a role in the FLASH effect. Montay-Gruel et al. [19] and Vozenin et al. [20], on the other hand, suggested that the FLASH effect might be driven by reduced reactive oxygen species.

The long-term effects of FLASH are not yet well-known; FLASH is a new treatment approach, and thus, not many patients have been treated with the new techniques [21]. As new studies are conducted and current research advances, the long-term effects, if any, will become evident within a decade or two [22,23].

### 1.2. Current FLASH Delivery

Most FLASH biological studies are performed using electron beams, as it is relatively easy to convert a conventional electron beam on a linear accelerator (LINAC) to a FLASH electron beam [24,25]. Although similar conversion techniques can be applied to photon beams, it is highly unlikely that an increased dose rate of 40 Gy/s could be achieved in a photon beam on a conventional LINAC, due to the low Bremsstrahlung production efficiency and the unlimited range of photons. As a result, new photon delivery systems need to be developed for delivering photon FLASH treatments [26,27].

Proton RT offers a convenient way for delivering FLASH treatments, as most modern proton units have the potential to produce proton beams with a much higher dose rate than that used in conventional proton treatment [28,29]. For example, the instantaneous dose rate of a modern proton beam with proton pencil beam scanning (PBS) capability [30] can be as high as several tens of Gy/s [28,31].

Transmission or shoot-through proton FLASH-RT [32,33,34,35,36,37,38] was the first delivery technique proposed for achieving FLASH dose rate using proton beams. In this technique, the highest energy proton beam is used, and the treatment target is positioned in the plateau (or entrance) instead of the Bragg peak region of the depth dose for this beam. Since the plateau region has a relatively homogeneous dose distribution, the whole target can be uniformly irradiated with the FLASH dose rate. This FLASH proton delivery technique is generally available to modern proton units with PBS capability [39,40]. However, since the Bragg peak occurs outside the patient body, there will be an additional exit dose beyond the treatment target, and the advantage of proton use for tissue sparing is lost.

### 1.3. Bragg Peak Proton FLASH

Although the implementation of transmission proton FLASH is straightforward for modern proton units, it has an obvious drawback, i.e., it trades the normal tissue sparing effects of the Bragg peak for that of the FLASH effect. Investigators have started to develop new delivery methods that utilize the Bragg peak instead of the plateau region for target positioning in order to to take advantage of both the Bragg peak and FLASH biological sparing effects.

For example, Kang et al. [41,42] have developed a conformal Bragg peak FLASH technique using a single-energy pristine Bragg peak. In this technique, the Bragg peak of each pencil beam is positioned at the distal end of the treatment target, and the delivery time for each pencil is carefully optimized to maximize the dose rate for every point along the path of each pencil beam. For a target that does not have the same depth at the distal end, a universal range shifter and range compensator can be used, ensuring that the Bragg peak will fall at the distal end of the target [41].

### 1.4. Optimization of Beam Parameters for Proton FLASH

Given the complexity of FLASH delivery, the beam parameters need to be optimized to maximize the dose rate. The impact of treatment planning and machine characteristics on the shoot-through proton FLASH was investigated by van de Water et al. [43], who concluded that multiple planning parameters, including beam energy, beam intensity, spot-wise intensity, and fractional dose need to be considered to achieve FLASH dose rates. van Marlen et al. [32] also published a treatment planning study on proton FLASH shoot-through therapy for normal fractionation head and neck cancer. The study concluded that a higher dose rate can be achieved using a smaller spot time, higher spot MU, and a higher gantry current.

Treatment planning studies have been reported for Bragg peak proton FLASH delivery using a universal range shifter and range compensator [41]. Wei et al. [44] developed an in-house treatment planning system that incorporated spot placement, weighting, and dose rate optimization through the inverse algorithm. This technique has been successfully applied to treatment planning studies on Bragg peak proton FLASH-RT for lung [44,45], head and neck [46], liver [47], and breast [48] cases.

We hypothesized that the Bragg peak FLASH technique could be applied in the treatment of prostate patients to achieve conformal radiation therapy, potentially reducing toxicity and costs. Thus, we investigated the dosimetric characteristics of Bragg peak proton FLASH-RT for prostate cancers. Using the in-house treatment planning system mentioned earlier [44], we retrospectively replanned for 10 prostate patients previously treated with proton PBS SBRT to 40 Gy in five fractions. A comparison of the resulting dose metrics between conventional PBS and Bragg peak FLASH plans will be presented in this paper, as well as the dose rate histogram (DRVH) and FLASH metrics of 40 Gy/s coverage (V_40Gy/s_). Finally, the potential of Bragg peak proton FLASH for prostate treatment will be discussed.

## 2. Materials and Methods

For conventional proton RT, multiple Bragg peaks at different depths together create a Spread-Out Bragg Peak (SOBP) or uniform dose region, so the cumulative peak encompasses the entire tumor. Contrary to this, the proton Bragg peak FLASH method uses a singular pristine Bragg peak that directly interacts with the tumor. Ten prostate patients who were previously treated at New York Proton Center (NYPC) using pencil beam proton therapy had their treatment replanned for using the proposed Bragg peak FLASH-RT. The treatment planning was performed using an in-house developed tool based on open-source software [49] that reoptimized the beam delivery parameters to meet clinical goals and achieve the highest FLASH dose rate coverage. BP treatment planning uses only the highest energy 250 MeV proton beam from the cyclotron. There was no energy degradation in the beam line to generate lower energy beams, and thus the high proton transmission efficiency and beam current were preserved to reach a FLASH dose rate. The universal range shifter (URS) and beam-specific range compensators (RCs) were used to adapt the Bragg peaks distally so that the exit dose was eliminated. In this study, the Bragg peak width (80% to 80%) is ~10 mm in water [45] and allows for a more efficient dose delivery than SOBP since the beam energy does not need to be varied.

### 2.1. Bragg Peak Planning Optimization

All 10 patients were previously treated using a stereotactic body radiotherapy (SBRT) regimen to deliver a 40 GyRBE dose in five fractions. The BP FLASH treatment planning used the same fractionation scheme. The CTV volumes ranged from 29.0 cc to 81.29 cc with a median of 55.8 cc. The spot spacing range was 5–8 mm. To improve the dose rate, a spot map optimization method was used to reduce the number of spots, so that the minimum MU of each field was increased to maintain a higher nozzle current in the ProBeam system [42]. The minimum MU changes from 250–300 for FLASH treatment planning, which is much higher than the minimum MU of one in the conventional dose rate. Once the data were generated with the optimal parameters, the plan was normalized so that at least 95 percent of the treatment volume would receive the prescription dose for both conventional PBS and Bragg peak plans. In the conventional proton PBS planning, two lateral fields shooting from gantry angles of 90 and 270 degrees were most commonly used. Each field delivered a uniform dose distribution to the target with a 50% beam weight. This two-beam arrangement was not used for BP FLASH planning due to single-energy layer delivery requiring more fields to achieve a uniform dose distribution. After investigating various planning approaches, a four-beam arrangement similar to the conventional two-beam arrangement was adopted for this study, with two lateral beams two degrees apart from the lateral incorporated in order to increase the flexibility of the single-energy Bragg peak technique. The FLASH treatment plans were optimized to meet clinical goals and compare to the conventional PBS treatments.

### 2.2. Ultra-High Dose Assessment

In proton PBS delivery, the field dose is delivered spot by spot. The dose rate calculation differs significantly from conventional flood field techniques, such as double scatter proton and electron therapy [50,51]. In double scatter proton therapy, the entire field receives a nearly uniform mean dose rate at the same depth. The localized dose rate calculation in a volume needs to take the spot delivery sequence into consideration. Currently, there are variable dose rate calculation methods for regions of interest (ROIs), including dose averaged dose rate (DADR) [43], dose threshold dose rate (DTDR) [42], and average dose rate (ADR) [52]. Neither DADR nor DTDR consider the spot delivery and scanning time for dose rate calculation, representing an instantaneous dose rate. ADR is a more conservative dose rate quantification method that includes spot delivery and scanning time for a localized voxel of ROIs. ADR is a quasi-mean dose rate method, and it was used in this study to assess the ultra-high dose rate coverage for targets and OARs. Like the dose volume histogram (DVH), a dose rate volume histogram (DRVH) was used to analyze the “FLASHness” of ROIs [45]. A FLASH ratio of V_40Gy/s_ represents the volume ratio receiving a dose rate of at least 40 Gy/s. It is utilized in this study to assess the potential FLASH sparing effect, as it reflects how much of the OARs can receive doses at a rate exceeding the 40 Gy/s threshold.

## 3. Results

### 3.1. Dosimetry Performance of the Bragg Peak Plans

**Figure 1:**
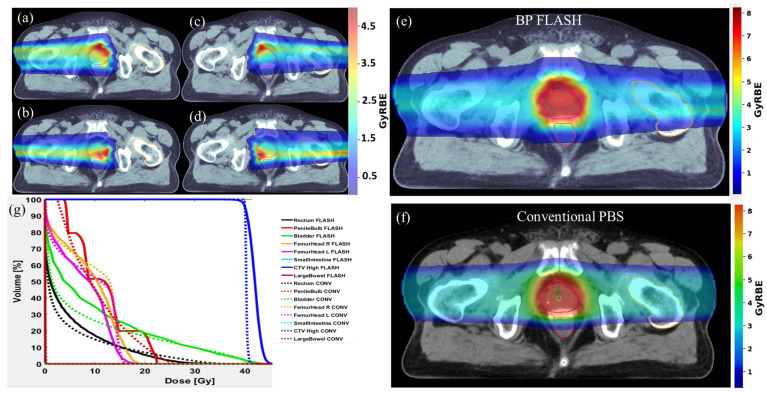
Using single-energy Bragg peak distal tracking to achieve conformable FLASH-RT in prostate cancer. (**a**–**d**) show the dose distribution of each field at gantry angles of 272, 268, 88, and 92 degrees, and (**e**) is the summed final dose distribution from all four fields. (**f**) is the 2D dose distribution of the conventional PBS plan using two opposing beams from the lateral direction. (**g**) is the DVH comparison between FLASH and conventional PBS plans.

A typical beam arrangement for Bragg peak treatment planning is shown in Figure 1a–d, where the arrows display the actual beam angles. As depicted in Figure 1a–d, each field covered only a partial part of the target, and all exit doses were eliminated by using the range-pulling back device, URS, and RCs. Summing up the doses from all four fields achieved a uniform distribution, as shown in Figure 1e. This patient had a CTV volume of 44 cc and was treated with proton SBRT using two lateral beams, and the composite dose distribution is shown using dashed lines in Figure 1g. It should be noted that the FLASH plan exhibited a less uniform dose distribution in the entrance part before reaching the target. This was because the Bragg peak plans used only a single-energy layer, and each spot delivered a minimum MU of 300. The DVH shown in Figure 1f confirms that the target coverage was the same, but with a slightly larger area receiving a higher dose inside the target (still within 15% of the prescribed dose). The critical OARs, namely the rectum, bladder, and penile bulb, exhibited comparable DVHs, particularly at the higher dose levels. Both the right and left femur heads received similar DVHs between the Bragg peak and conventional PBS plans. All the rest of the OARs, including the small and large bowels, were far from the target area and received relatively low doses, meeting clinical metrics.

Each of the 10 patients was optimized with the same clinically used beam arrangement in Bragg peak FLASH treatment plans. The desired dose metrics for treatment, listed in Table 1, were compared for both FLASH and conventional PBS. The results for the 10 involved patients can be seen in Table 1. A Wilcoxon signed-rank test was conducted comparing Bragg peak and conventional PBS, and a *p*-value less than 0.05 indicated a significant difference between the two techniques. Regarding the dose constraints for OARs, there were no significant differences between the two techniques except for the maximum dose metric for the urethra and bladder and the V_20Gy_ of the bladder. Similarly, due to the lack of optimization flexibility, including far fewer spots and higher MUs from each spot, the CTV showed slightly less uniformity in the Bragg peak plans compared to the conventional PBS plans, with a *p*-value < 0.05, indicating a significant difference.

### 3.2. Ultra-High Dose Rate Characteristics

**Figure 2:**
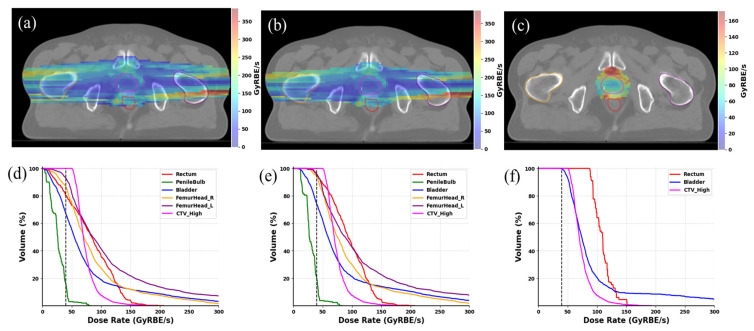
Ultra-high dose rate distribution for the selected patient. In (**a**–**c**), the 2D dose rate distribution is depicted by applying dose thresholds of 0.1, 1, and 5 Gy to the composite planned dose in a single fraction delivery. In (**d**–**f**), the DRVHs for all CTV (CTV) and OARs are presented by applying dose thresholds of 0.1, 1, and 5 Gy, respectively.

The ultra-high dose rate distribution was evaluated. Figure 2 shows the 2D dose rate distribution for the composite plan (a–c) with 3 different dose thresholds as well as the DRVH (h) from the same representative case in Figure 1. The voxel-based dose rate calculation includes the spot scanning and delivery time. The scanning pattern follows a zigzag fashion, with the faster scanning magnet in the primary scanning direction (y-direction). The results exhibit a higher dose rate at the field edge and a lower dose rate in the central area. This pattern is observable through the peak and valley dose rate distribution depicted in the color map. In plan optimization, the principle of “as low as reasonably achievable” (ALARA) was adhered to, with all OARs receiving minimal doses, not accounting for any additional FLASH sparing effect. All the OARs received only minimal doses without considering the additional FLASH sparing effect. The rectum, being a critical organ located in close proximity to, or having overlap with, the target’s high dose region, can receive significant doses. Hence, it has clinical significance to assess the FLASH dose rate ratio through the application of various dose levels. Three dose thresholds were considered to assess the ultra-high dose rate ratio for OARs. As shown in Figure 2a–c, higher dose thresholds can exclude low dose voxels from the 2D dose rate map. Accordingly, the V_40Gy/s_ coverage was increased for all of the OARs, as displayed in Figure 2d–f. When only considering voxels that received more than 5 Gy, the 5 Gy isodose lines do not touch most OARs, except for a small portion of the bladder and rectum volume close to the target, as displayed in DRVH of Figure 2f. This shows that the BP FLASH method can provide high conformity and minimize the doses to all the critical OARs.

**Figure 3:**
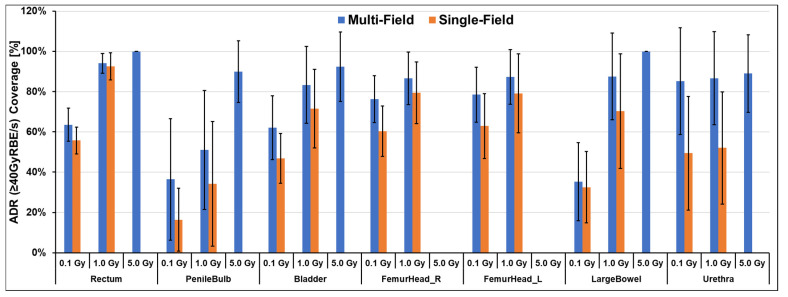
Ultra-high dose rate ratio V_40Gy/s_ for all the major OARs. The V_40Gy/s_ values are presented under the applied dose rate thresholds for both composite beams and single beams. Each bar corresponds to the averaged V_40Gy/s_ values across all 10 patients, while the whiskers indicate the standard deviation.

The dose rate distributions were quantified by applying 0.1, 1, and 5 Gy dose thresholds for individual fields and composite plan doses. The dose rate statistics for all prostate patients were averaged to quantitatively evaluate the coverage of FLASH dose rate, and the results are shown in Figure 3. Considering dose thresholds for each field is equivalent to assessing the ultra-high dose rate coverage field by field, which aligns with current biological studies using single-field delivery. Displayed in Figure 3, the V_40Gy/s_ ratios exhibited variation corresponding to dose thresholds. Higher FLASH ratios were reached when higher dose thresholds were taken into account. Upon the application of the 1 Gy dose threshold, the V_40Gy/s_ ratio increased to >70% for OARs, including the rectum, bladder, femoral heads, and large bowels, during single-field evaluation. With the use of an 8 Gy dose per fraction, which is relatively low compared to the 5 Gy dose thresholds, only low doses were delivered to each OAR, and no dose exceeded 5 Gy in single-field delivery. When the 5 Gy dose threshold was applied to composite doses, all OARs demonstrated a V_40Gy/s_ > ~90%. In the case of the rectum, the FLASH ratio nearly reached 100% after applying the 5 Gy threshold.

## 4. Discussion

In this study, we investigated the feasibility of prostate Bragg peak FLASH SBRT. Using a single-energy 250 MeV beam, we demonstrated that the dose metrics of the Bragg peak FLASH plans were comparable to the conventional PBS plans for most OARs. Similar CTV coverage was also achieved with the Bragg peak FLASH plans, except that the FLASH plans resulted in a higher D_max_ than the clinical plans. FLASH dose rates were achieved for most OARs, as the delivery time can be significantly reduced without energy switching. This FLASH dose rate of 40 Gy/s potentially provides extra OAR sparing benefits when compared to conventional methods due to the high dose rate. These results show that the single-energy Bragg peak method is clinically feasible for prostate SBRT treatment to further improve OAR sparing.

For high doses delivered to small CTVs, clinical PBS plans showed superior dose uniformity compared to FLASH plans, which is consistent with previous findings in lung [45], liver [47], head-neck [46], and breast [48] cancer cases. Conventional PBS treatment plans rely on multiple energy layers, small weighting spots, and a denser spot map to achieve planning goals. However, the BP FLASH method utilizes only the highest energy layer of the delivery system, compromising optimization possibilities in the dimension of energy layers. To enhance the delivery efficiency of the Bragg peak FLASH beam while preserving a >40 Gy/s dose rate, the minimum MU for each spot was substantially increased to between 250 and 300 in comparison to ~1 MU adopted by conventional PBS plans. This resulted in a high dose being delivered in each individual spot. Furthermore, the number of spots was intentionally reduced during optimization of the Bragg peak plans for increased delivery efficiency. However, this reduction in spot count compromised on flexibility to achieve a more uniform dose distribution in comparison to conventional PBS treatment plans. During the optimization of Bragg peak plans, we also allowed a higher maximum dose within the CTV to ensure that all constraints of critical OARs dose metrics were met, as shown in Table 1. Specifically, we ensured that the D_max_ of the penile bulb was less than 100% of the prescription, and the D_max_ was not higher than 105% for other neighboring OARs like the rectum.

Most current biological studies use a single field, e.g., transmission FLASH proton beam to deliver a full prescribed dose to the target area, demonstrating the FLASH sparing effect for normal tissues. While the Bragg peak FLASH technique in this study uses multiple fields, each field only covers a partial volume of the target and OARS. Although plan optimization aims to achieve as much FLASH dose rate coverage for the volume of each field of irradiation, further biological studies are needed to investigate if the FLASH sparing effect still exists with this delivery approach. As evidenced, the ultra-high dose rate FLASH biological protection effect is determined not only by dose rate, but also by the dose that the tissue received [53,54]. As shown in Figure 3, the FLASH dose coverage is much higher if we only consider the volume that receives 5 Gy, as opposed to the 0.1 Gy or 1 Gy thresholds. At present, the optimal dose threshold, beyond which the additional OAR sparing effect of FLASH effect is clinically significant, is yet to be determined. We expect that this threshold is in the order of a few Gy for prostate SBRT with 8 Gy daily fractional doses. Future clinical trials are necessary to establish safety and effectiveness regimens for Bragg peak application.

We would like to emphasize that all studies relating to Bragg peak FLASH SBRT are based on beam parameters of clinically available machines and on current clinical regimens, which provide a useful reference for future clinical trials and applications. Single-energy Bragg peak plans with a highly conformal dose distribution can be delivered using commercially available PBS proton machines with some adjustments. First, the only additional hardware required are the URS and RCs, which have been widely used in scattering and PBS delivery techniques. Therefore, no significant challenges in manufacturing and quality assurance (QA) are expected, due to our familiarity with them in proton therapy. Second, to advance this novel technique, we will need vendor support to integrate URS and RCs into the commercial nozzle design to support single-energy delivery. Finally, this method needs to be implemented into commercial treatment planning systems (TPS) so that additional treatment studies can be conducted to explore the potential and limitations of this delivery technique.

In this study, even without assuming any FLASH sparing effect, the outstanding dosimetry characteristics of the single-energy Bragg peak highlight the potential applications of this technique in prostate SBRT. There are at least two major benefits that can potentially impact current proton therapy. First, the typical beam-on time can be reduced for each beam from 1 min to less than 1 s per field. This also eliminates the potential migration of the prostate gland during beam-on under Bragg peak FLASH delivery, providing more accurate fiducial alignment and facilitating easier fiducial marker tracking. For critical OARs in prostate cancer treatment, the rectum is notably important. Using spacer OAR is a more widely accepted standard practice in prostate treatment; it helps to distance the rectum from the high-dose region, and thus allows for increased dose escalation. Considering the Bragg peak FLASH technique’s conformity to conventional RT, coupled with the presumed FLASH sparing effect for OARs, challenges related to motion control and image-guided radiation therapy (IGRT) may be reduced. In addition, the single-energy Bragg peak technique represents a more efficient delivery method, as there is no need for variable energies from the beamline. Therefore, the energy selection system and beam focusing components can be eliminated from cyclotron-based proton systems, making proton systems more compact. This reduction in space and cost could significantly enhance the affordability of proton therapy.

## 5. Conclusions

This study demonstrated the feasibility of using the Bragg peak FLASH technique using a clinical proton machine for prostate SBRT, following a clinical regimen of 40 Gy in five fractions. The results of this treatment planning study showed that the single-energy Bragg peak FLASH delivery technique can achieve comparable plan quality to the conventional PBS technique while maintaining adequate FLASH dose rate coverage. Moreover, this innovative Bragg peak FLASH technique eliminates exit dose with greater conformity compared to the shoot-through proton FLASH technique. Further biological and clinical studies are needed to validate the clinical implications of this novel delivery technique.

## Figures and Tables

**Table 1:** Dosimetric comparison between the Bragg peak FLASH-RT and conventional PBS treatment plans for prostate SBRT treatment. All dose metrics utilized the median and first and third quartile (Q1 and Q3) values across all 10 patients.

Target and OARs	Metric	Conventional PBS	Bragg Peak FLASH	*p*-Value
CTV	D_max_ (%)	103.5 (103.0, 103.7)	117.0 (115.4, 118.3)	0.005
Femur Head_L	D_max_ (cGy)	1731.0 (1689.5, 1742.3)	1964.5 (1871.5, 2092.6)	0.059
	V 2160 cGy (cc)	0 (0)	0 (0)	0.317
Femur Head_R	D_max_ (cGy)	1720.3 (1705.6, 1736.0)	1956.5 (1824.8, 2096.7)	0.059
	V 2160 cGy (cc)	0 (0)	0 (0)	0.180
Large Bowel	D_max_ (cGy)	189.5 (67.8, 1970.4)	861.1 (164.5, 2095.1)	0.241
Penile Bulb	D_max_ (cGy)	1928.2 (1263.7, 2809.0)	1983.0 (1338.8, 2784.4)	0.721
	D 3 cm^3^ (cGy)	209.0 (142.6, 1851.0)	346.1 (203.4, 1884.8)	0.139
Rectum	D_max_ (cGy)	3856.5 (3634.9, 3955.2)	3935.5 (3703.6, 4113.9)	0.386
	Dmean (cGy)	374.6 (298.3, 422.7)	103.1 (94.4, 389.3)	0.445
	D 1 cm^3^ (cGy)	3019.7 (2661.8, 3310.4)	3121.5 (2932.9, 3327.1)	0.953
	V 2400 cGy (%)	4.3 (3.2, 5.7)	5.0 (3.9, 7.9)	0.285
	V 3015 cGy (%)	1.6 (0.6, 2.9)	1.7 (0.7, 2.7)	0.575
Small Intestine	D_max_ (cGy)	5.3 (2.0, 22.5)	5.3 (5.0, 16.7)	0.401
Urethra	D_max_ (cGy)	4041.7 (4031.5, 4051.8)	4436.3 (4418.6, 4491.0)	0.005
Bladder	D_max_ (cGy)	4071.0 (4065.0, 4089.0)	4456.9 (4333.3, 4564.6)	0.008
	V 3600 cGy (%)	5.0 (2.7, 5.7)	2.9 (2.2, 5.2)	0.374
	V 2000 cGy (%)	13.1 (8.8, 13.8)	13.0 (11.5, 15.1)	0.021

## Data Availability

Data are contained within the article.
